# Food Neophobia in Wild Rats (Rattus norvegicus) Inhabiting a Changeable Environment—A Field Study

**DOI:** 10.1371/journal.pone.0156741

**Published:** 2016-06-02

**Authors:** Klaudia Modlinska, Rafał Stryjek

**Affiliations:** Institute of Psychology, Polish Academy of Sciences, Warsaw, Poland; University of Lethbridge, CANADA

## Abstract

Food neophobia is a reaction to novel food observed in many animal species, particularly omnivores, including *Rattus norvegicus*. A neophobic reaction is typically characterised by avoidance of novel food and the necessity to assess both its potential value and toxicity by the animal. It has been hypothesised that this reaction is not observed in rats inhabiting a changeable environment with a high level of variability with regard to food and food sources. This study was conducted in such changeable conditions and it aims to demonstrate the behaviour of wild rats *R*. *norvegicus* in their natural habitat. The rats were studied in a farm setting, and the experimental arena was demarcated by a specially constructed pen which was freely accessible to the rats. At regular intervals, the rats were given new flavour- and smell-altered foods, while their behaviour was video-recorded. The results obtained in the study seem to confirm the hypothesis that rats inhabiting a highly changeable environment do not exhibit food neophobia. The observed reaction to novel food may be connected with a reaction to a novel object to a larger extent than to food neophobia. The value of the results obtained lies primarily in the fact that the study was conducted in the animals’ natural habitat, and that it investigated their spontaneous behaviours.

## Introduction

Food neophobia involves avoidance of novel foods [1, 2]. It is found in many animal species—see e.g. [3–7]. The need to differentiate between edible and inedible foods is observed, to a high degree, in omnivores, and the problem these animals encounter while searching for food is referred to as the omnivore’s/generalist’s dilemma [8]. Many animal species do not consume unfamiliar foods, and this tendency may persist up to several days and become reinforced in a new environment [9–11]. An animal which encounters novel food cannot discern whether the food is edible or not. In its reaction to food novelty one can discern two components. First, after noticing a novel object, an animal has to overcome the fear of novelty and assess the properties of the object. Then, it has to determine the consequences of consuming such unfamiliar food (food neophobia). This behaviour typically involves initial avoidance of the novel food, followed by gradual sampling of the new food at certain intervals [2]. When coming in contact with novel food, the rat extends its neck towards it, inspects it with whiskers and sniffs. Then, it takes a small food sample and moves away from the container. If the new food is not associated with adverse bodily reactions, the consumption of such food increases [12]. Any food which elicits an illness in the rat within several hours, becomes aversive to the animal [13, 14]. Numerous studies on neophobia have hitherto been carried out on the wild rat (*Rattus norvegicus*). Research shows that a neophobic reaction to novel food is observed both in wild and laboratory rats—e.g. [12, 15]. However, the changeability of the environment inhabited by the rat may result in an avoidance reaction of varying degrees depending on the different categories of changes occurring in such environment.

It has been suggested that the emergence of food neophobia was influenced by human attempts to eradicate rats from human surroundings [5, 16–18]. Those species of rats which, due to the independence of their diet from human activity, have not been exposed to population control measures, such as poisons, do not exhibit neophobic reactions to novel foods [5, 16, 17, 19]. In addition, food neophobia seems not to be elicited in rats inhabiting landfill sites, where the environment is subject to constant change and novelty [20, 21]. No neophobic reactions were observed in studies on a *Rattus norvegicus* population which had inhabited an island isolated from human influence for over one hundred years [18]. However, in their study, Taylor and Thomas [18] mainly evaluated the effectiveness of disinfestation methods; they did not focus on the issue of food neophobia. Furthermore, their measurement of the reaction to novelty was based on the observation of bait intake, but not on a detailed observation of the animals' response to food.

Based on the above-mentioned studies, it may be hypothesised that environment changeability forces omnivores to lower their neophobia threshold and to consume foods of unknown properties. A detailed analysis of an animal’s behavior in contact with new food may show not only the absence or presence of food neophobia, but also its level, or individual components of such a reaction.

The purpose of the experiments outlined below was to investigate reactions to novel foods in rats inhabiting a constantly changing environment in which they could find different sources of diversified foods. Based on previous studies and hypotheses formulated by researchers working on food neophobia [5, 16–19], we hypothesise that in such conditions rats will not exhibit food neophobia or that the intensity of neophobic reactions will be insignificant.

Because much of the research on this issue has so far been carried out on laboratory rats, we decided to conduct our studies on wild rats. Several comparative studies indicate that wild rats differ considerably from their laboratory counterparts–both in terms of behaviour and with regard to their morphology and physiology [1, 22–30]. The large number of domestication-induced changes which laboratory rats undergo is the reason why a high degree of caution should be applied when using laboratory rats as study objects [23–24, 26, 29, 31–32]. Therefore, the choice of the wild rat as the object of studies on natural behaviours seems to be well-grounded.

Additionally, it seems that best-suited for investigating such phenomena is the animals’ natural habitat, in which natural behaviours in wild-living individuals are investigated. Although such experimental conditions fail to allow researchers to control many variables, the degree of ecological validity of this type of studies is often much greater [33]. This is essential, as the conclusions drawn from the study may be extrapolated to other populations of the species under investigation, and thus, to a potential application significance of the study results—see e.g. [32, 34]. In addition, the animals observed while exhibiting spontaneous behaviours in a familiar environment seem exposed to a much lower level of stress than animals in laboratory studies, which is particularly important in the context of animal welfare.

In the present study, two experiments were conducted on reactions to novel food in changeable environment. The experiments took place on a private farm, inhabited by a wild-living colony of *R*. *norvegicus*. The purpose of the first experiment was to assess the utility of the place and method for observing ingestive behaviours of rats. It allowed us to undertake preliminary observation of the rats' reactions to novel foods and to plan the next experiment. The aim of the second experiment was to conduct a precise quantitative measurement of the reaction to novel foods. This involved a re-arrangement of the experimental arena. A new procedure was implemented to enable the researchers to estimate the differences in the rats' behaviour when the animals encountered familiar and novel foods, all other study parameters being equal. More variables were recorded as compared with Experiment 1, which enabled a more complete statistical analysis.

## Material and Methods

### Ethics statement

Under Polish law, the above-described studies did not require permission of the local ethics committee for animal experimentation, as it was a field experiment conducted on a pest species. It involved only a slight environmental rearrangement and providing animals with non-poisonous food. None of the above caused any harm to animals.

The study was carried out on private land with the permission of the owners.

### Animals

This study was conducted on a free-living colony of brown rats (*Rattus norvegicus*) on a farm situated on the outskirts of Warsaw, Poland. Prior to the start of the experiment, the wild rat colony had been observed for six months by means of camera traps. In addition, analyses had been undertaken of the distribution of rat burrows, paths, the number and distribution of rat droppings, the amount of bait taken, etc. It was done in order to assess the size, distribution and condition of the population. On the basis of the data collected and the data from a previously conducted study [32], the size of the rat population had been estimated at approx. 50 individuals. The size of the colony had not been controlled for 4 years prior to the experiment–neither by means of poisons, nor mechanically. The animals had been living in highly diversified dietary conditions–they fed mainly on foods which had been temporarily stored in the barn (fruits, vegetables, crops) and on the highly diversified food they found in the nearby compost container, on horse feed (the horse was fed hay and, occasionally, bread and oats), and on the highly diversified dog food given to dogs which lived in the adjacent rooms. The physical environment was characterised by a certain degree of changeability–the owner of the buildings made frequent modifications by displacing hay and emptying the compost container; in the process he systematically discovered new corridors and rat nests. Furniture and agricultural equipment stored in the buildings were also frequently moved from one place to another.

### Indoor pen

**Fig 1:**
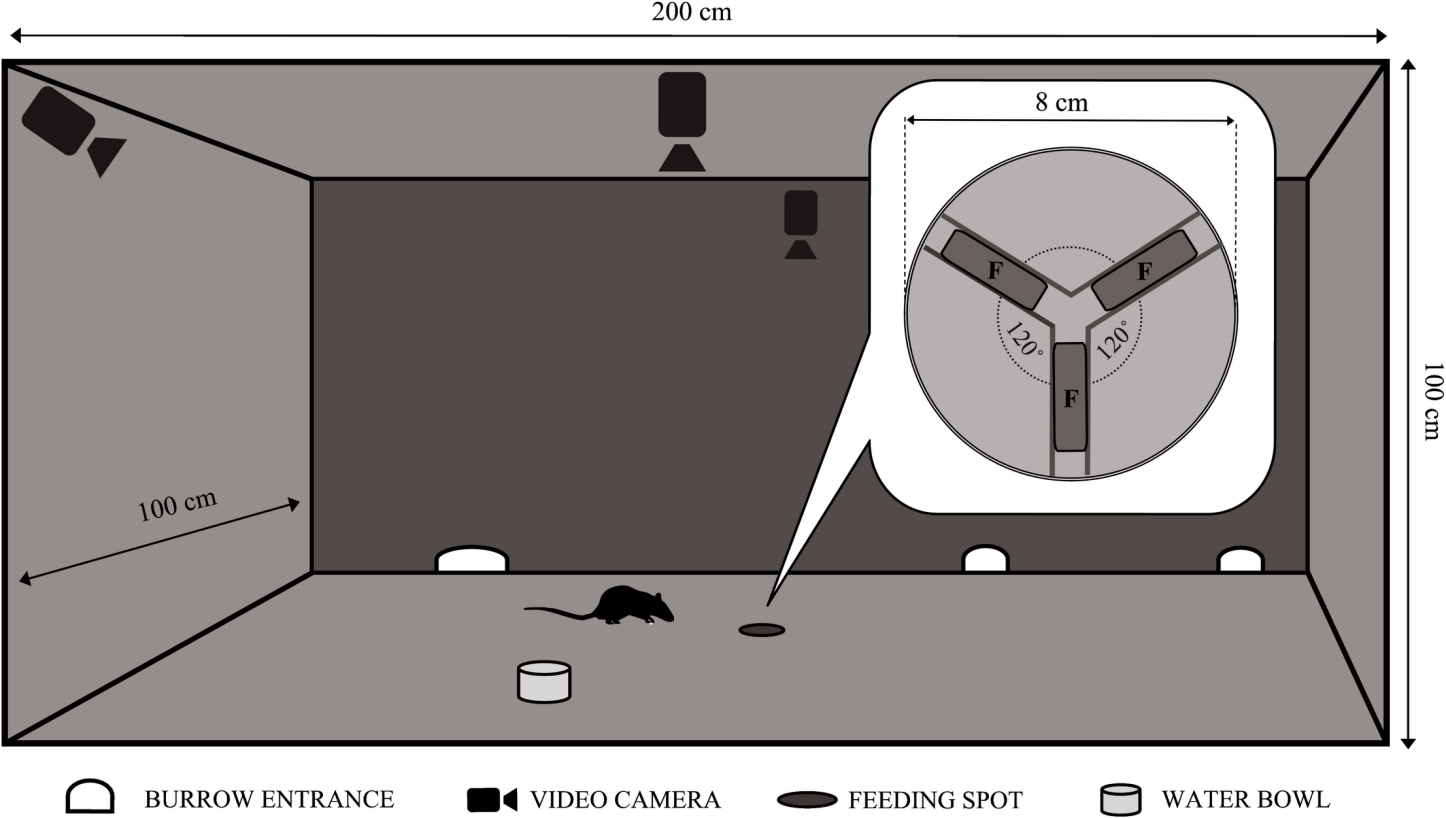
View of the pen with the location of the bowl in which the food was placed. The detail shows a magnified outline of the bowl with the distribution of the food pellets (F).

**Fig 2:**
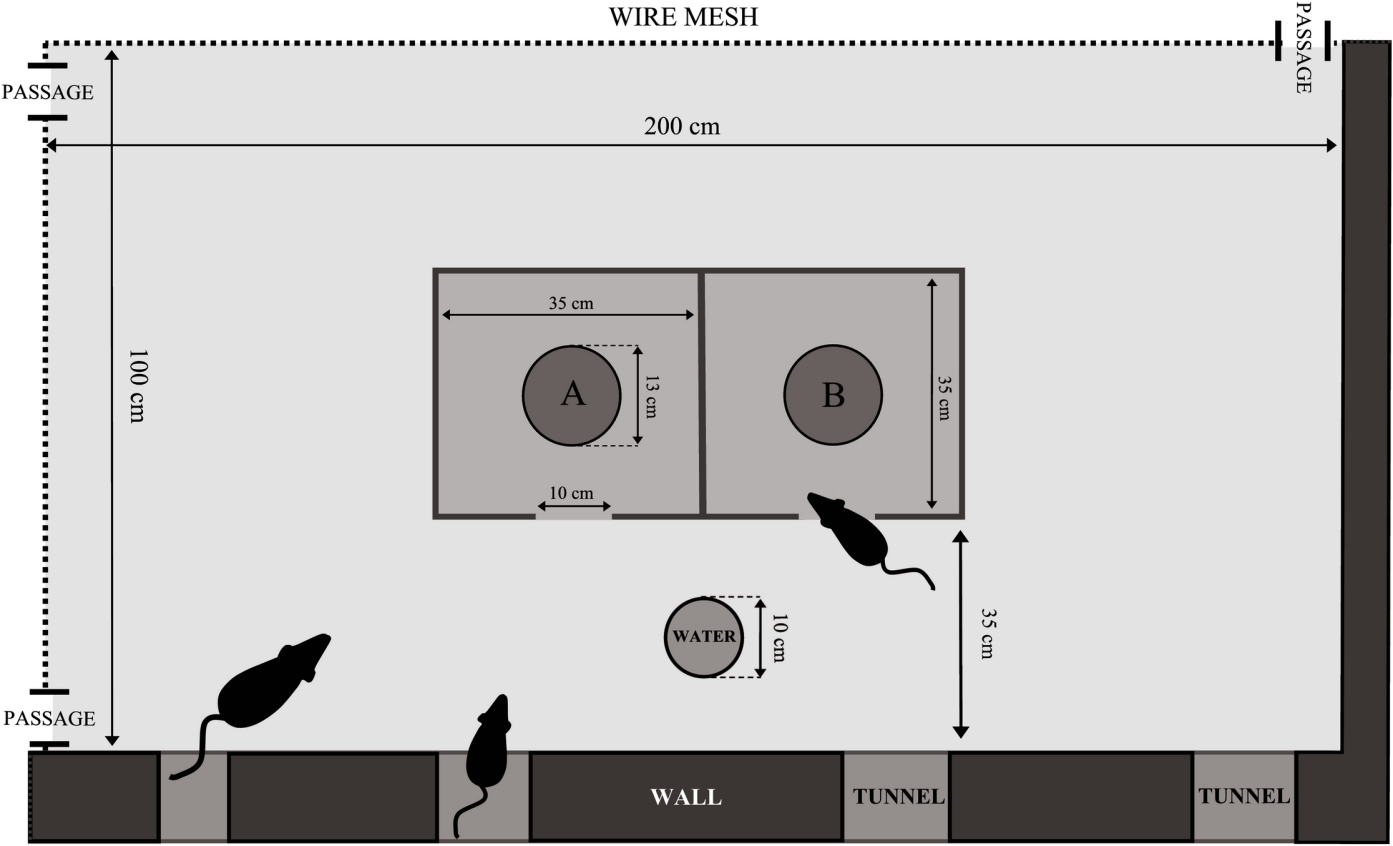
An outline of experimental area. A and B indicate bowls in which food pellets were placed on a daily basis.

Rat behaviour was observed in one of the buildings located on the farm, in a pen which had been fenced specifically for this purpose (see Figs 1 and 2 below in the descriptions of experiments). The pen (200cm/100cm/100cm) was located in the stable, adjacent to the barn. To prevent predator-induced variability of rat behaviour [35] we used dog- and cat-proof indoor pen fenced with wire mesh. We used 3–4 IR cameras connected to a DVR recorder, which enabled 24/7 observation bouts. The rats could access the pen easily through 6 to 9 entrances–the number of entrances (of which 3 to 5 were entrances leading directly from the rats’ underground burrows to the pen) varied in time. The nests were situated in the adjacent room. Light intensity during day-time was low ranging from a maximum of 20lx in the box to a maximum of 50lx in the remaining part of the pen.

Humans did not enter the pen during the experiments described. All objects, feed and water were supplied and put on the ground through a lid on top of the pen. Water was provided ad libitum. To minimise the influence of human scent, which could affect the rats’ behaviour, disposable nitrile gloves were always used by the staff.

### Experiment 1

The first experiment was carried out as preliminary observation of reactions to novel foods in wild living colony of *Rattus norvegicus*.

#### Procedure

The observation took place in autumn 2015 (from September 9 until October 12). The average daily temperature in the pen was 12°C (min. 2°C, max. 22°C). The times of sunrises ranged from 6:00 a.m. at the start of the experiment to 6:55 a.m. at its end. The times of sunsets ranged from 7:06 p.m. to 5:49 p.m.

A bowl (see Fig 1), 8cm in diameter, was placed at the centre of the indoor pen. To prevent the bowl from being displaced, it was fixed to the ground with a long nail, driven in through the centre of the bowl. The inside of the bowl was inlaid with double strips of odourless glue, which were arranged at an angle of 120° and used to keep the food which was put in the bowl in place (Fig 1). The bowl was filled with laboratory rat feed (Labofeed H, WP Morawski, Kcynia, Poland) in the form of pellets, ca. 20mm long, 12mm in diameter, and 3,5g +/-0,5g in weight. The animals were accustomed to this type of feed, as it had been used for 4 months as means of monitoring the presence and size of the rat colony. Each day of the experiment between 6 and 8 p.m. (i.e. just before the peak of rats' circadian activity–for reference see [26]) three food pellets were placed in the bowl (Fig 1). After a 4-day-long habituation period, the researcher changed the flavour and smell of one of the three pellets supplied on a daily basis. This was done by moistening the pellet with water and coating it with one of the powdered spices. The following were used (in the order as indicated): cinnamon, nutmeg, peppers, caraway, and turmeric. These spices had been used in other studies to mark the novelty of a given food—e.g. [15, 36–39]. As these spices are neither bitter nor hot in taste, it was assumed that they would not be aversive to the animals investigated. The new flavour pellets were randomly put in one of the three available places (Fig 1). Standard pellets were put in the remaining two places. The procedure with the use of the new flavour was repeated for 2 consecutive days. The flavoured pellet was each time moved to the next available position in the clockwise direction. To prevent habituation of the colony to continuous novelty, the periods when the new flavoured food was supplied were separated by intervals of 2-3 days during which only the standard feed was supplied.

#### Results

We conducted a binomial test of the null hypothesis that the frequency of taking the new pellet is random (i.e. probability of picking each pellet equals 0.33) [40]. On the first day on which the new flavoured food was supplied (N = 5) the new food was the last to be picked by the rats, while in one instance the new flavoured food was not picked at all (p = 0.004). The flavoured pellet was picked last (p<0.001) in 11 out of 15 performed exposures of flavoured pellets. In 2 instances the flavoured pellet was not picked at all.

The difference in time that lapsed between the rats picking the first and the second pellet did not diverge significantly from the difference observed between the second and the third (flavoured) pellet–p>0.05.

### Experiment 2

The purpose of the second experiment was to conduct a precise quantitative measurement of the reaction to the novelty of the food in a wild living colony of *R*. *norvegicus*. This involved a re-arrangement of the experimental arena and a modification to the procedure. These changes were implemented to enable the researchers to estimate the differences in rat behaviour when the animals encountered familiar and novel foods, all other study parameters being equal. More variables were recorded as compared with Experiment 1, which enabled a complete statistical analysis.

#### Apparatus

A box built from OSB (Oriented Strand Board), with external dimensions of 74cm x 37.5cm, and wall height of 40cm (see Fig 2), was placed at the centre of the indoor pen. The box was comprised of two independent compartments with single entrances (10cm x 10cm) located on the same wall of the box. A black rubber bowl (13cm in diameter and 1,5cm high) was placed in each of the compartments (A and B—Fig 2). The bottom of the boxes was covered with earth which was identical to the surface of the remaining part of the experimental arena (clayish sand); the earth layer was ca. 5-10mm thick.

#### Procedure

The experiment took place in winter 2015/2016 (from December 4 until January 18–46 days in total). The average daily temperature in the pen was 4°C (min. -10°C, max. 8°C). The times of sunrises ranged from 7:22 a.m. at the start of the experiment to 7:36 a.m. at its end. The times of sunsets ranged from 3:27 p.m. to 3:57 p.m.

In the course of the experiment, the exact same feed was used as in Experiment 1 (Labofeed H, WP Morawski, Kcynia, Poland). The animals were fed at approx. 3 p.m. (i.e. before the peak of the rats' activity).

During the first 6 days of the experiment, 15 standard pellets of laboratory feed that the rats were familiar with were put in the bowls (A and B) located in the boxes; the purpose of this was to habituate the animals to the new procedure.

Following the habituation period, 15 flavoured pellets were placed in the bowl in one of the compartments. This time the researchers also made use of spices (the following were used: cocoa, cardamom, allspice, curry, and winter savory). The spices had again been selected on account of their non-toxicity and potentially non-aversive reaction in rats, while, in addition, the spices that had been selected were different from the spices used in Exp. 1. After the pellets had been moistened with water, they were coated in one of the above-mentioned spices. 15 standard flavour pellets were put in the other adjacent compartment, which served as a control measurement. The procedure with the new flavour was repeated for 5 consecutive days. To prevent habituation to continuous novelty, every period of five days when novel food was supplied was followed by 3 days during which the animals were given the standard feed in both compartments. The new flavoured pellets were supplied alternatively in the right-hand and left-hand compartments, to avoid the place effect. After the period of supplying a given flavour was over, in order to eradicate any traces of smell, the earth from the bottom of the box was replaced in both compartments (the experiment and the control compartments) and new bowls were put in.

The experimental area was equipped with 4 IR cameras connected to a DVR recorder, which enabled 24/7 observation bouts. The following variables were calculated on the basis of video-recordings: the latency to pick individual pellets (calculated from the moment the rats entered the particular compartment), the number of instances where rats approached the food without picking the pellet (calculated until the last of the 15 pellets was picked), as well as the duration and frequency of the periods spent in the left and right part of the box. Only those behaviours were taken into consideration that were recorded during periods from the moment the pellets were supplied until 8 a.m. on the following day.

#### Results

The study was conducted on a colony of wild rats in their natural environment. The animals were not marked. The lack of possibility to assess the behaviour of individual rats was partly offset by the number of measurements performed (over a period of 46 days), the considerable size of the colony and by a qualitative analysis.

The data was interpreted by means of non-parametric statistical tests. Differences were considered significant for p values of ≤ 0.05.

Two points of reference for the reaction to novelty were used: cross-sectional (comparison of the rats' behaviour in the compartment with novel food and in the compartment with standard fodder); and longitudinal (analysis of the response to food novelty after a period of access to standard fodder).

Cross-sectional analysis. In the initial phase of the analysis, a comparative examination was conducted of the rats’ behaviour in the experiment compartment (where new food was supplied) and in the control compartment (where familiar food was provided).

No differences were demonstrated in the behaviour of rats between compartments with regard to the pace at which pellets were picked, measured as the length of time that elapsed between the instances of picking individual pellets (U = 114.50; p = 0.197). In addition, no differences were observed with respect to the time the rats spent in each of the box parts (U = 224.00; p = 0.396).

Differences were observed between the number of times the rats approached novel and familiar food without picking the pellet (Mann-Whitney U test–U = 125.00; p<0.001). The rats approached the container with food, sniffed, and then moved away from the food without picking the pellet much more frequently in the case of novel food (mean rank 32.29) than in the case of familiar food (mean rank 18.00).

Based on Mann-Whitney U test, the median latency to pick all pellets from the control compartment diverged significantly from the median latency to pick all pellets from the experiment compartment (U = 147.00; p = 0.006). The mean rank for the control compartment was 18.88, while for the experiment compartment it was 29.82. The differences in latency to pick pellets between the compartments were also observed at consecutive stages. There was a difference between the median latency to pick the first 5 pellets (U = 155.50; p = 0.002), 10 pellets (U = 156.50; p = 0.012) and 15 pellets (U = 147.00; p = 0.006).

Longitudinal analysis. Next, a comparison was conducted of the animals’ behaviour when confronted with novel food at subsequent stages of the experiment. The factor taken into consideration was the rats’ reaction in the initial phase of novel food provision (the first 3 days during which novel food was supplied). The reaction was compared to the rats’ behaviour in the preceding three-day period when no novel food was provided and the animals were given the standard feed in both compartments.

**Fig 3:**
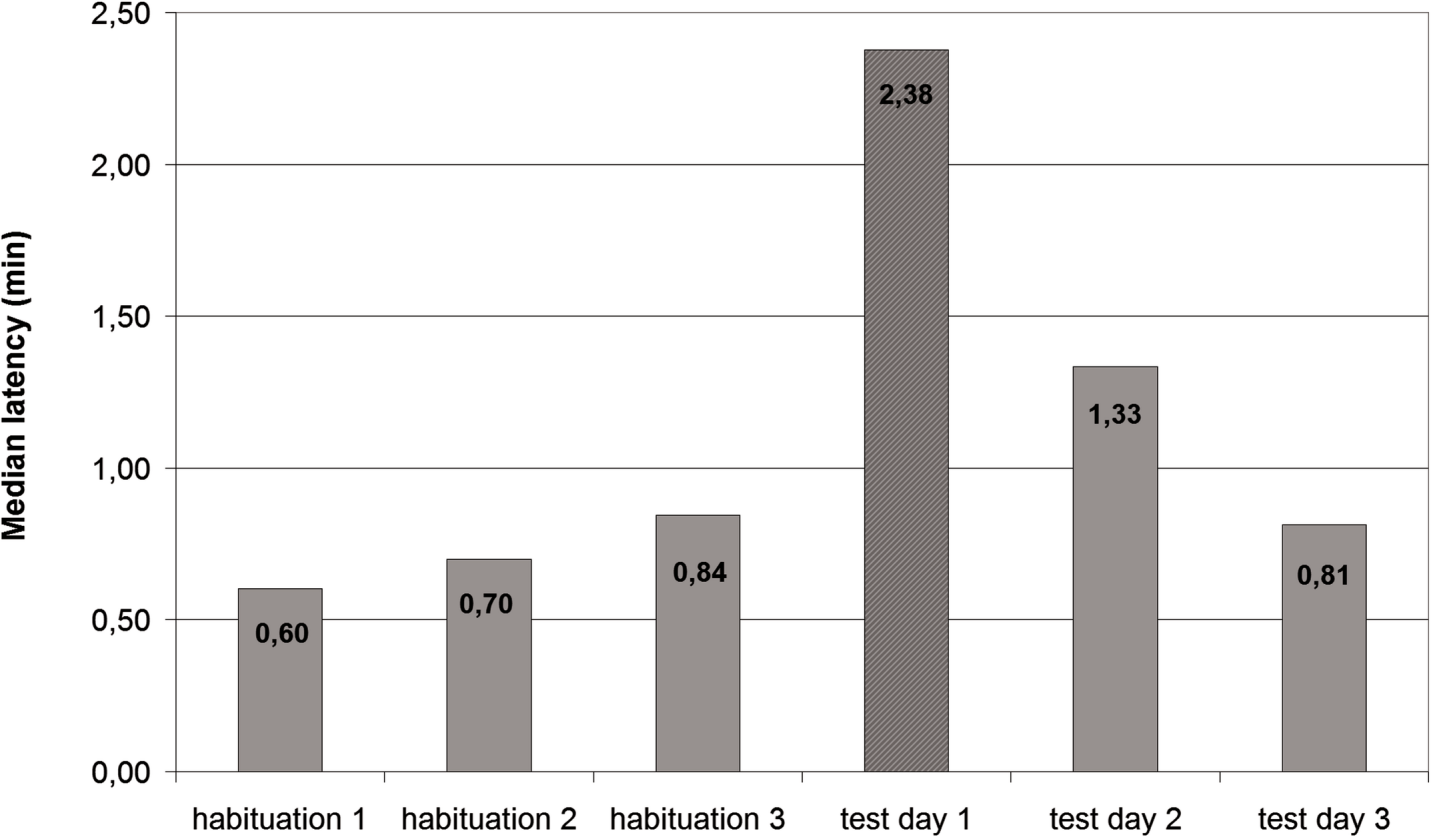
Median latency (the decimal logarithms of median values) of picking food off novel food pellets on consecutive days of the experiment.

The median latency to pick novel food was a time-varying parameter; the Friedman test value was χ2(5, N = 5) = 11.629; p = 0.04)–Fig 3. A detailed analysis by means of Wilcoxon test showed that this variability resulted from an increase in the median latency on the first day when novel food was supplied (Z = -2.023; p = 0.043). On the third day of the experiment, however, the latency level did not differ from the level observed during the habituation sessions (Z = -0.944; p = 0.345).

A significant increase in latency was observed particularly in the case of latency to pick the first new flavoured pellet (Friedman test– χ2(5, N = 5) = 20.778; p = 0.001). Wilcoxon test showed that a considerable increase in latency occurred on the first day following the supply of novel food (Z = -2.023; p = 0.043).

Differences at the subsequent stages of the experiment were observed also with regard to the number of instances where the rats approached novel food without picking the pellet (Friedman test– χ2(6, N = 4) = 17.153; p = 0.004). A detailed analysis by means of Wilcoxon test demonstrated a significant increase in the number of times the rats approached food on the first day when novel food was provided (Z = -2.060; p = 0.039). However, as soon as on the second day when novel food was supplied, the number of instances where rats approached food did not diverge considerably from the level observed during the habituation sessions (Z = -1.633; p = 0.102).

None of the rats consumed the food on the spot. The food which had been picked by a rat from the container was always taken away from the box. In two instances, not all new flavoured pellets were picked by the rats before the recording time was over (that is, before 8 a.m.). In one instance, this occurred on the first day when novel food was supplied (cardamom), in another instance–on the forth day when novel food was supplied (curry). However, during that time, the rats came neither to the compartment where familiar food was placed, nor to the one where novel food was put; it is therefore difficult to state whether the reason the pellets were left intact was the new flavour or some other event that took place outside of the pen area which might have influenced the rats’ behaviour.

The box was entered by individuals of both sexes and of different ages. No regular activity pattern was observed. The composition of the groups which entered the pen also differed, as did the times at which the animals appeared in the pen.

## Discussion

An analysis of the results obtained in the experiments showed that the introduction of novel food was noticed by rats and elicited a behavioural response. In the first preliminary study, in most cases the novel food was the last to be picked by the rats. However, no significant differences were observed with regard to the time that elapsed between individual pellets were picked. It means that the length of time that elapsed between a standard pellet and the flavour pellet was picked was not greater than the time that elapsed between the points at which standard pellets were picked. In the second experiment, in which a comparison was conducted of the rats’ behaviour in the two parts of the box, in one of which novel food was supplied, while familiar food was placed in the other, differences were observed in the behaviour of the same animal group. The rats approached novel food and investigated it much more frequently than in the case of familiar food. The familiar food to which the animals had been habituated, was mostly picked without any observable investigatory behaviour. Also, the latency to pick all food pellets was higher in the case of novel food, while the increase in latency was most pronounced on the first day when novel food was supplied. The first day on which novel food was supplied was also characterised by a considerable increase in the number of instances the rats approached the food without picking the pellet.

The above results show that wild living rats exhibit a neophobic reaction to the novelty of the food. Contact with a novel food elicits an exploratory reaction in rats and results in an increase in exploratory behaviour (measured as the number of instances where rats approached novel food and sniffed it), but this reaction may be connected to a larger extent with a reaction to the novelty of the object's properties than with food neophobia as such. In the second experiment, in most instances (that is, on 23 out of 25 days) the rats picked all new flavoured pellets, and continued to pick pellets on subsequent days. What is more, as early as on the third day when food was supplied, the level of latency to pick pellets dropped to the level observed in the habituation sessions. A similar tendency was observed with regard to the number of times the rats approached novel food without picking the pellet; as early as on the second day of the experiment, this level was comparable to the level in the habituation sessions. In addition, habituation to food of novel properties may be linked to its non-toxicity and to the fact that the food was not aversive to the rats under investigation.

The novel food was given to the rats in a familiar place and in a familiar container, which enabled us to eliminate the effect of the novelty of the place and the novelty of the container, an effect which is sometimes disregarded in certain studies—e.g. [1]. The only change the rats encountered was the altered flavour and smell of the feed. In the theory of reactions to novel foods, food novelty involves neophobia which is linked to the novelty of the object and to the necessity to assess whether a given object is edible, as well as food neophobia, which results in cautiousness when consuming a new food which might prove toxic—e.g. [2, 8, 20]. Based on the results obtained in the studies outlined above, we may presume that we managed to observe this first stage of reaction to novel food, that is, neophobia linked to the novelty of the object. No characteristic symptoms of food neophobia were observed, such as sampling the novel food before picking the pellets or a lower pace at which the new food was picked—cf. [15]. Furthermore, no interval was observed between the consumption of the first pellets and the continued consumption of subsequent pellets, which might have suggested fear of the potentially harmful effects of consuming a new food—cf. [1].

The rats under investigation inhabited an environment characterised by a high degree of changeability, both with regard to the surrounding objects, and the available foods. The considerable size of the colony, and the resulting competition for particular resources, may have induced the animals to exploit the different food sources available in their immediate surroundings. This changeability and diversity of foods, unintentionally provided by humans, coupled with a motivation to obtain food from different places may have lead to habituation to food novelty, particularly as the rats were not exposed to any danger, such as poisons used in the course of disinfestation.

The results of the experiments outlined in this paper seem to support the hypothesis that rats inhabiting a highly changeable environment do not exhibit food neophobia [20, 21]. In addition, the results are also in line with those obtained in studies by Taylor and Thomas [18], who observe no food neophobia in a wild living rat colony not exposed to pest control. Their research method did not allow them to assess the initial reaction to the novelty of the food, as the respective study focused solely on whether the rats picked the food or not. Nevertheless, it appears that similar conclusions may be drawn from the results of this study, as in most cases the rats picked all the pellets, including the novel ones, before the night was over.

In addition to confirming the hypothesis about the lack of symptoms of food neophobia in rats inhabiting a changeable and safe environment (lack of danger of pest control), the results of this study clearly demonstrate the rats’ behaviour when they come in contact with novel food for the first time. It may be suggested that the authors of the study succeeded in isolating behaviours exhibited by animals when encountering the novelty of an object, associated with a new food, even in the case of a low level of food neophobia.

Nonetheless, it is difficult to assess the magnitude of habitat changes for the rats. Although we selected the place of the study due to the complexity and variability of its environment, we can not be sure whether this level of changeability was a significant factor influencing the animals' behaviors. In the near future, we plan to conduct further experiments involving different levels of environment changeability, which may deepen the understanding of this issue.

The value of the results obtained lies primarily in the fact that the study was conducted in the species’ natural habitat. Furthermore, the study investigated spontaneous behaviours exhibited by these animals—cf. [41]. The experimental conditions may have enabled a reduction in stress levels experienced by the animals, which is a significant variable in research on wild rats in laboratory settings—see [15, 42]. A high level of fear in wild rats, probably resulting from low adaptation levels to the laboratory environment is a factor in favour of field studies. Moreover, substituting wild rats with laboratory rats in studies investigating animal behaviour further undermines the reliability of generalising the results obtained in this study to other rat populations due to considerable behavioural and morphological differences between wild and laboratory rats [1, 21–23, 25–27, 29–30, 43–44]. Bearing the above considerations in mind, one may presume that the shortcomings of field studies (such as lack of full control over environmental conditions, impossibility of marking or identifying individuals, etc.) should not be regarded as contraindications to conducting research in the form of field studies. This is particularly true if, as in the case of the above-outlined study, the object of investigation is a large population, and the colony distribution and environmental conditions have been investigated prior to the start of the experiments.

## Supporting Information

S1 Dataset (CSV)
